# Epicardial slices: an innovative 3D organotypic model to study epicardial cell physiology and activation

**DOI:** 10.1038/s41536-021-00202-7

**Published:** 2022-01-17

**Authors:** D. Maselli, R. S. Matos, R. D. Johnson, C. Chiappini, P. Camelliti, P. Campagnolo

**Affiliations:** 1 Faculty of Health & Medical Sciences, School of Biosciences & Medicine, Section of Cardiovascular Sciences, University of Surrey, Guildford, GU2 7XH UK; 2 Centre for Craniofacial and Regenerative Biology, King’s College London, SE1 9RT London, United Kingdom

**Keywords:** Heart stem cells, Heart stem cells, Regenerative medicine, Tissue engineering

## Abstract

The epicardium constitutes an untapped reservoir for cardiac regeneration. Upon heart injury, the adult epicardium re-activates, leading to epithelial-to-mesenchymal transition (EMT), migration, and differentiation. While interesting mechanistic and therapeutic findings arose from lower vertebrates and rodent models, the introduction of an experimental system representative of large mammals would undoubtedly facilitate translational advancements. Here, we apply innovative protocols to obtain living 3D organotypic epicardial slices from porcine hearts, encompassing the epicardial/myocardial interface. In culture, our slices preserve the in vivo architecture and functionality, presenting a continuous epicardium overlaying a healthy and connected myocardium. Upon thymosin β4 treatment of the slices, the epicardial cells become activated, upregulating epicardial and EMT genes, resulting in epicardial cell mobilization and differentiation into epicardial-derived mesenchymal cells. Our 3D organotypic model enables to investigate the reparative potential of the adult epicardium, offering an advanced tool to explore ex vivo the complex 3D interactions occurring within the native heart environment.

## Introduction

The epicardium plays a crucial role in the embryo during heart development^1^. Lineage tracing studies indicate that the embryonic epicardium is a major source of cardiac fibroblasts^2^, cardiac adipose tissue^3^, vascular smooth muscle cells, and pericytes of the coronary vasculature^4^. In response to injury, such as myocardial infarction (MI), the adult epicardium is able to retrace the embryonic gene expression, upregulating transcription factors such as *Wilms’ tumor 1* (WT1) and *T box 18* (Tbx18)^5–7^. In this context, epicardial cells undergo epithelial-to-mesenchymal transition (EMT) and start migrating into the myocardium, contributing to remodeling, re-vascularization, and repair through differentiation and paracrine stimulation^8,9^. Many studies described the epicardial reactivation as an evolutionarily conserved mechanism, but its efficiency varies greatly among different species: from the ability to regenerate significant portions of the adult heart in lower vertebrates, to a restricted reparative window during the early days after birth in small mammals^10^. Despite some clinical evidence of the human newborn hearts’ recovery capacity^11^, the role of the epicardium in human adult heart repair has been largely investigated through primary or progenitor-derived cell culture experiments^12–14^. These valuable systems do not however replicate the multicellular complexity of the tissue and the interaction with the extracellular matrix (ECM), which play a critical role in the epicardial-mediated repair^15^. Therefore, a robust and comprehensive model of the epicardial physiological environment is needed to help unveil the adult epicardial cell potential in large mammals.

Epicardial activation resulting in enhanced heart repair can be achieved experimentally by priming the heart with thymosin-β4 (Tβ4)^9,16^. Tβ4 is a 43 amino-acid long peptide that enhances the innate epicardial response following MI via epigenetic regulation of *WT1* promoter^5^ and drives epicardial EMT, ultimately increasing neovascularization and reducing pathological remodeling^5,17,18^. In the context of post-MI fibrosis, Tβ4 reduces collagen deposition both by attenuating profibrotic gene expression^19^ and by resolving the immune response, as demonstrated in vivo in zebrafish and mouse models of cardiac injury and in vitro on human monocytes cultures^20^. Adenoviral overexpression of Tβ4 also displayed a therapeutic potential in a pig model of chronic myocardial ischemia by mediating capillary growth and maturation^21^. Further studies and new models are required to investigate the pleiotropic role of Tβ4 in post-MI repair, in particular in large adult animals.

In this study, we developed a translational model, which employs swine hearts to investigate the role of endogenous and exogenous stimuli on the regulation of adult epicardium. Our innovative cutting protocol preserves the epicardium intact, allowing us to produce viable tissue slices which contain both epicardium and myocardium and maintain the typical 3D organization of cardiac tissue which enabled the investigation of the interaction of the adult epicardium with its natural niche. Epicardial slices represent a new, inexpensive, and versatile ex vivo model to study the adult epicardium in large mammals.

Through culturing the epicardial slices ex vivo we were able to maintain a high viability rate for several days and recapitulate Tβ4-dependent epicardial activation, previously observed in murine models in vivo. Our results showed that, even in the absence of an ischemic stimulus, Tβ4 treatment resulted in epicardial cells activation leading to the overexpression of epicardial transcription factors and EMT markers in the slices, enhancing the epicardial cell motility and differentiation.

Our epicardial slice system exclusively enables to study the adult porcine epicardium in a complex 3D system with the versatility of an in vitro/ex vivo culture. In summary, we provide a unique and convenient research tool to dissect the role of the epicardium in cardiac homeostasis and repair in large animals, enabling further studies to harness the epicardial regenerative capacity for therapeutic purposes.

## Results

### Epicardial slices encompass the complete epicardium/myocardium interface

**Fig. 1:**
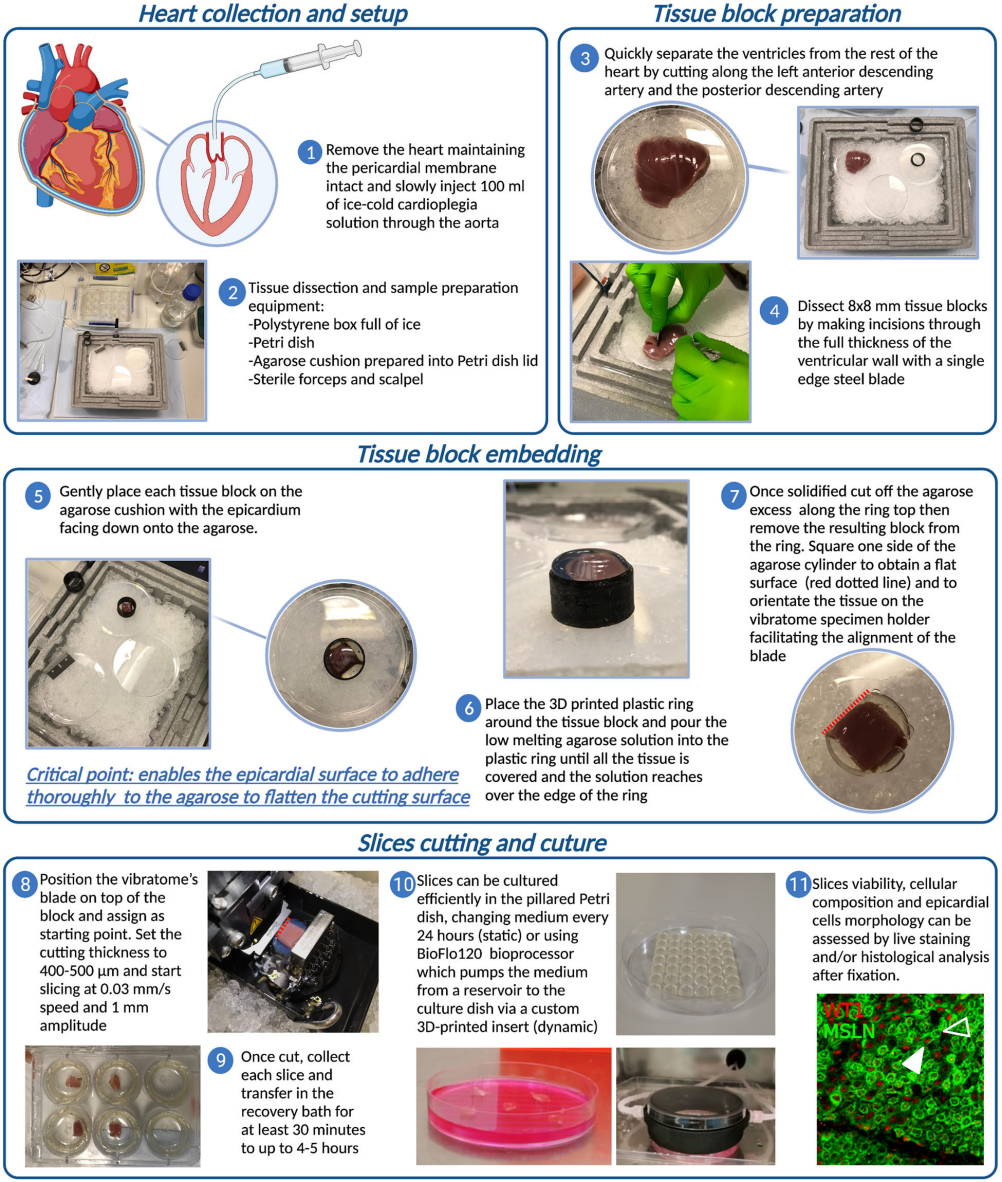
Porcine epicardial slices preparation. Schematic representation of porcine ventricles sampling and slicing. **Heart collection and setup:** (1) Heart is harvested maintaining the pericardium and retroperfused with ice-cold cardioplegia solution. Ventricles are then separated from the heart avoiding touching the epicardium, to prevent damage. (2) Embedding area must be set up prior to tissue slicing to allow all components to cool down to ice-cold temperature. **Tissue block preparation:** (3) Cardiac tissue is placed on the ice-cold Petri dish and (4) blocks of tissue 8 mm ×8 mm are dissected with a single edge steel blade. **Tissue block embedding:** (5) The epicardial side of the slice is flattened onto a cold agarose surface and placed in the middle of a custom-made 3D printed ring. 5–8 ml of 30–37 °C low melting agarose is poured to embed the tissue block. (6) Once solidified, the block is removed from the ring and (7) squared on one side to allow a better alignment with the vibratome’s blade (red dashed line). **Slice cutting and culture**: (8) Embedded tissue blocks are oriented along the squared side and glued to the vibratome specimen holder. The blade is aligned to the epicardial surface and 400–500 µm thick slices are cut using a high precision vibratome. (9) Slices are allowed to recover for at least 30 min in a recovery bath. (10) Epicardial slices are cultured epicardium-up on 8-mm-high pillars cast at the bottom of a Petri dish. (11) *En face* immunohistochemistry of WT1 (red) and mesothelin (MSLN, green) shows the presence of a continuous epicardial layer, characterized by typical morphology and marker expression (WT1^+^ cells indicated with full arrowhead, WT1^−^ MSLN^+^ cells indicated with empty arrowhead). This figure was created using BioRender.com.

**Fig. 2:**
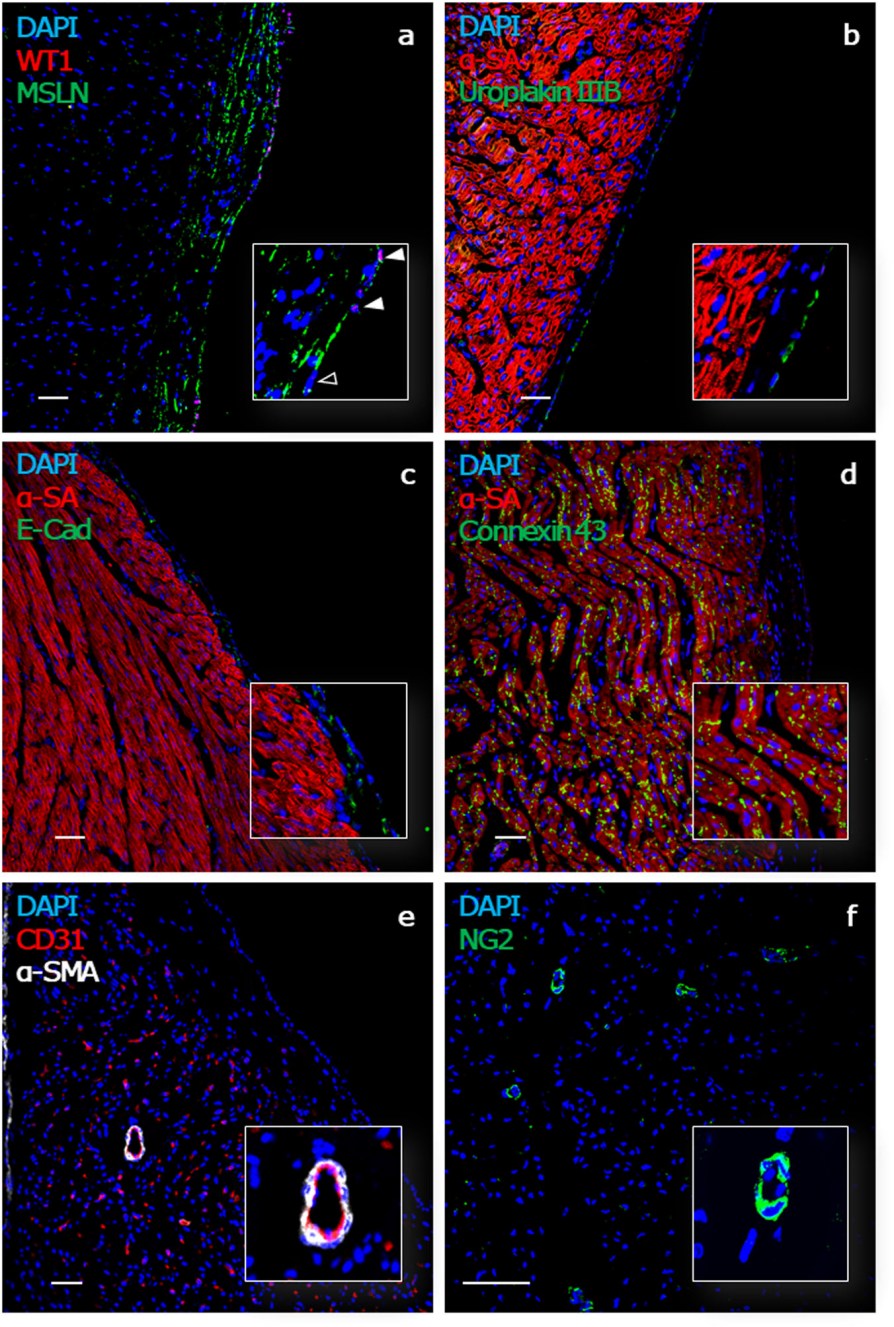
Epicardial slices characterization. Confocal microscopy analysis of formaldehyde-fixed OCT-embedded porcine epicardial slices confirms the presence of an intact epicardial layer identified by their localization and flat morphology and expressing WT1 and MSLN (WT1^+^ epicardial cell indicated with full arrowhead, WT1^−^ epicardial cell indicated with empty arrowhead) (**a**), the Uroplakin IIIB (**b**), and E-cadherin (**c**). The cardiomyocytes within the slice display α-sarcomeric actin structures and the expression of connexin 43 within the gap junctions (**d**), indicative of the preserved myocardial architecture. CD31 identifies vascular structures, including arterioles surrounded by smooth muscle cells expressing α-SMA (**e**). CD31^+^ capillaries, associated with NG2^+^ pericytes, are visualized in higher magnification images (**f**). Nuclei are labeled with DAPI. Scale bar, 50 µm.

Intact hearts were obtained from experimental or abattoir pigs and carefully flushed with cardioplegia solution (Fig. 1, step 1), before transportation. Once the heart is dissected into tissue blocks (Fig. 1, steps 2–4), the embedding process can begin. In standard protocols developed to obtain myocardial slices, the epicardial surface is often sacrificed to flatten the cardiac tissue and ensure the alignment of the myocardial fibers^22,23^. In order to maintain a viable epicardial cell monolayer, we developed an alternative protocol that protects the epicardium while ensuring the myocardium alignment. Here, heart blocks are embedded in low melting agarose, a technique which has successfully been tested previously on cardiac slices^24,25^. Differently, from previously published embedding protocols, the focus of our procedure is to preserve an intact epicardium by flattening the external (epicardial) layer of the block onto a compliant surface during the embedding (Fig. 1, steps 5–7). The leveled epicardial surface is then used to carefully set the starting position for the slicing process (Fig. 1, step 8). From each tissue block, using a high precision vibratome, we obtained 400–500 µm slices which were allowed to recover (Fig. 1, step 9) and then either processed for histological and molecular analysis or cultured on pillared plates, in static or dynamic conditions, and then subjected to further analysis (Fig. 1, step 10 and 11). Further details of the preparation and culture method can be found in the extensive Methods section of this manuscript. Structural analysis of histologically processed slices identified a single layer of epicardial cells and a sub-epicardial layer containing epicardial-derived mesenchymal cells, forming the outermost layer of the heart and expressing Mesothelin (MSLN) (Fig. 2a). Over 50% of the cells within the top epicardial layer expressed the transcription factor WT1 (57.06 ± 7.49% WT1^+^ epicardial cells, *N* of pigs = 8, *N* of slices = 8), with a distinctive nuclear localization (Fig. 2a). The epicardial marker Uroplakin IIIB was detected on a subset of MSLN^+^ cells in the epicardium (Fig. 2b and Supplementary Fig. 1), while a smaller number of cells expressed low levels of the epithelial marker E-cadherin (Fig. 2c). Immunostaining for α-sarcomeric actin (α-SA) and Connexin 43 indicated the retention of a structurally preserved and connected myocardium undelaying the epicardium, presenting organized contractile units (Fig. 2b–d) and intact gap junctions (Fig. 2d). In addition, the slices presented intact vascular structures characterized by the endothelial expression of CD31, including neuron-glial antigen 2^+^ (NG2) pericyte-covered capillaries and larger vessels surrounded by α-smooth muscle actin (α-SMA)-expressing smooth muscle cells (Fig. 2e, f).

Our characterization demonstrated that freshly cut epicardial slices presented an undamaged epicardial monolayer, covering the underlying connective and myocardial tissues. Therefore, this ex vivo model maintains the structural and cellular composition of the intact heart tissue, ideal premises to exploit it to explore the epicardial/myocardial interface ex vivo.

### Ex vivo culture maintains the integrity of epicardial slices

**Fig. 3:**
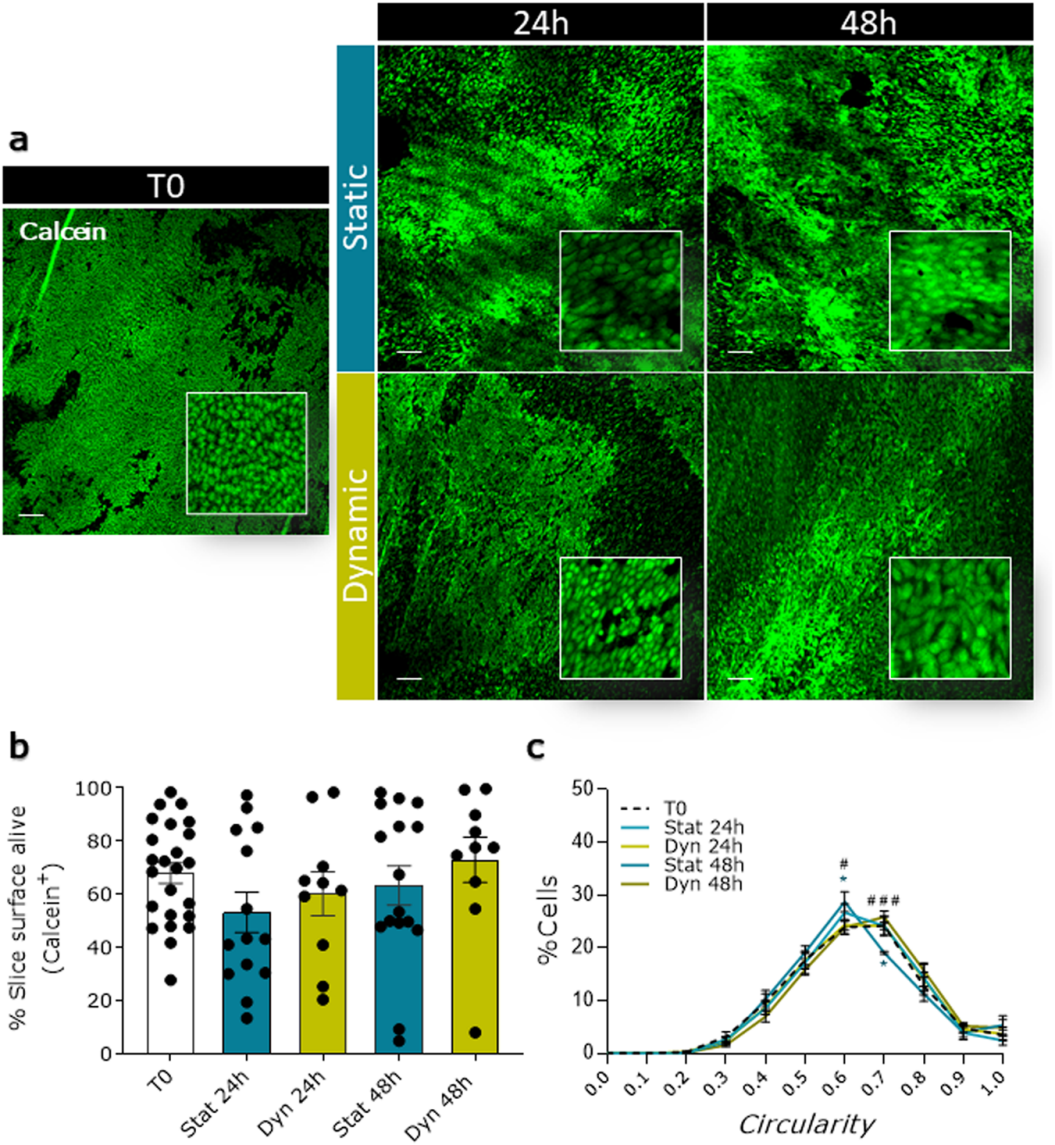
Slices viability and epicardial cells morphology over culture. Cell viability in freshly cut slices (T0) and after 24 and 48 h of static (Stat) and dynamic (Dyn) culture. The epicardium maintains its morphology and viability upon culture (**a**), as quantified by the average percentage of the calcein AM positive area (**b**) *N* of pigs = 6–18, number of slices displayed in the graph. Epicardial cells circularity distribution (**c**). *N* of pigs = 3–5, number of slices: T0 = 8, Stat 24 h = 5, Dyn 24 h = 6, Stat 48 h = 5, Dyn 48 h = 7. All graphs display data as mean ± SEM. *p ≤ 0.05 vs T0, ^#^*p* ≤ 0.05 Stat 48 h vs. Dyn 48 h, ^###^*p* ≤ 0.001 Stat 48 h vs. Dyn 48 h. Scale bar, 100 µm.

**Fig. 4:**
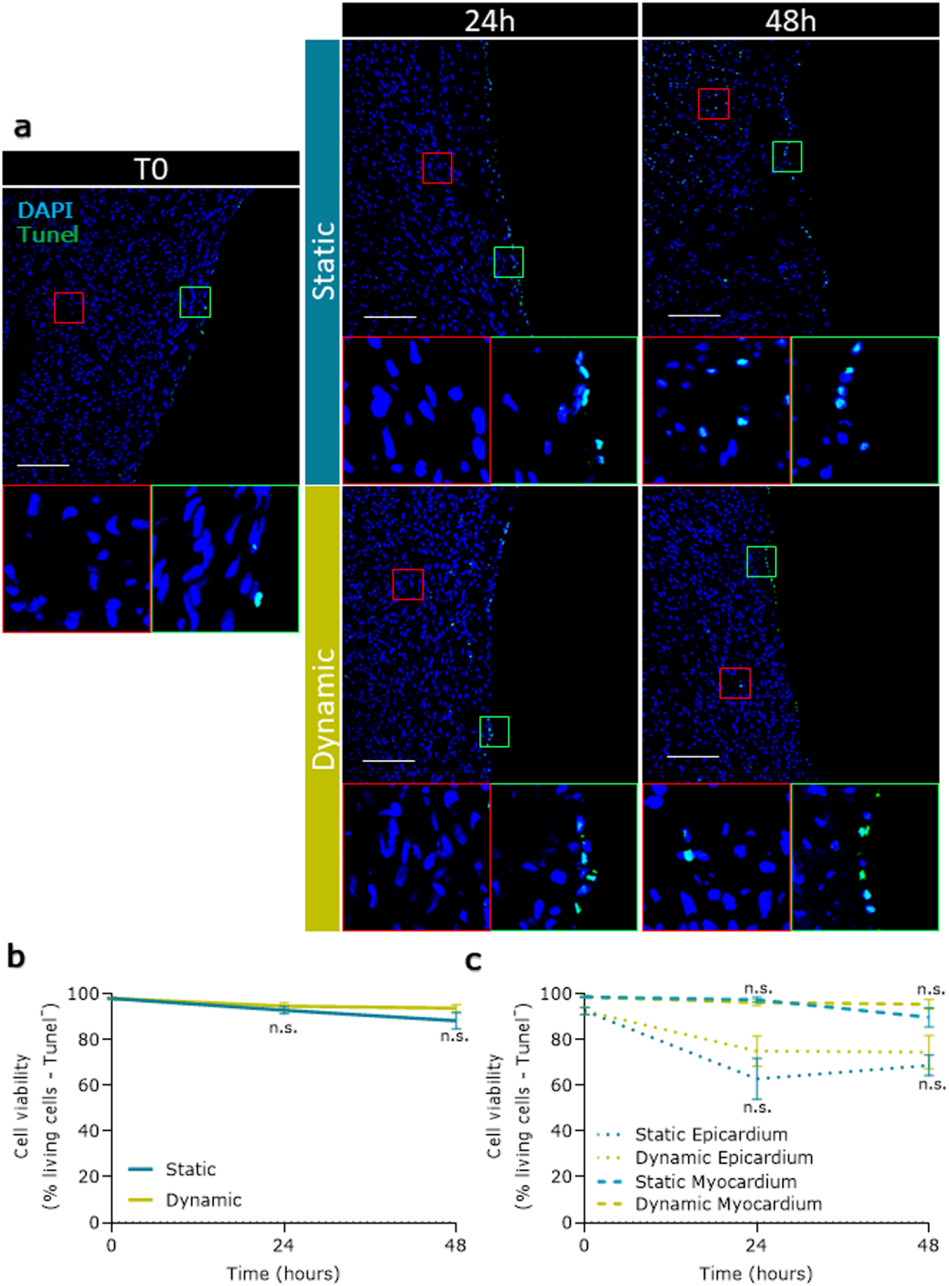
Apoptosis levels in cultured epicardial slices. Apoptotic cells were evaluated by ApopTag detection kit assay (**a**); graphs report a fraction of non-apoptotic cells as a percentage of the total nuclei in the slice (**b**) and independently calculated fraction for myocardium and epicardium (**c**). *N* of pigs = 3–6, number of slices: T0 = 13, Stat 24 h = 7, Dyn 24 h = 4, Stat 48 h = 7, Dyn 48 h = 5. All graphs display data as mean ± SEM. n.s. not significant vs. Static control. Scale bar, 100 µm.

We used two different ex vivo culture systems to maintain epicardial slices: a simpler and inexpensive “static” method and a more controlled and tunable “dynamic” method. The static method was based on a liquid–air system previously used to culture myocardial slices, which optimizes oxygen supply^26^. The dynamic method was based on a perfusion bioreactor system (flow rate 4 ml/min) equipped with a feedback loop control system capable of the real-time monitoring and adjustment of the dissolved oxygen level at 21% and the pH at 7.4 in the culture medium. We compared the static and dynamic systems by measuring cell viability, apoptosis, and proliferation rate after 24 and 48 h. Calcein acetoxymethyl (Calcein AM) staining was used to quantify the live/metabolically active cells on the epicardial surface of fresh slices (Fig. 3a). Confocal microscopy quantification of fluorescence showed that both static and dynamic cultures maintained the initial slice viability over a period of 48 h, with a remarkable viable area of over 50% throughout (Fig. 3b). In addition, cultured slices overall retained the characteristic cobblestone-like morphology of the epicardium in culture, confirming the presence of undifferentiated epicardial cells after 48 h of culture (Circularity mean ± SEM: T0 0.673 ± 0.016; Stat 24 h 0.661 ± 0.017; Stat 48 h 0.669 ± 0.013; Dyn 24 h 0.679 ± 0.010; Dyn 48 h 0.701 ± 0.009) (Fig. 3c). Histological analysis (Fig. 4a) revealed that freshly cut slices presented minimal levels of apoptosis and that the overall apoptosis was not increased in either type of culture (Fig. 4b). In the specific, the epicardium displayed moderate levels of apoptosis in both cultures, confirming the Calcein AM results and detecting a time-dependent adaptation to the culture in the first 24 h. Myocardium exhibited a remarkably low level of apoptosis throughout, confirming the suitability of our culture systems for the maintenance of viable epicardium and myocardium (Fig. 4c). Importantly, the myocardial expression of Connexin 43 and α-SA was maintained in both culture conditions, with the dynamic culture better preserving the organization of the structures (Supplementary Fig. 2).

**Fig. 5:**
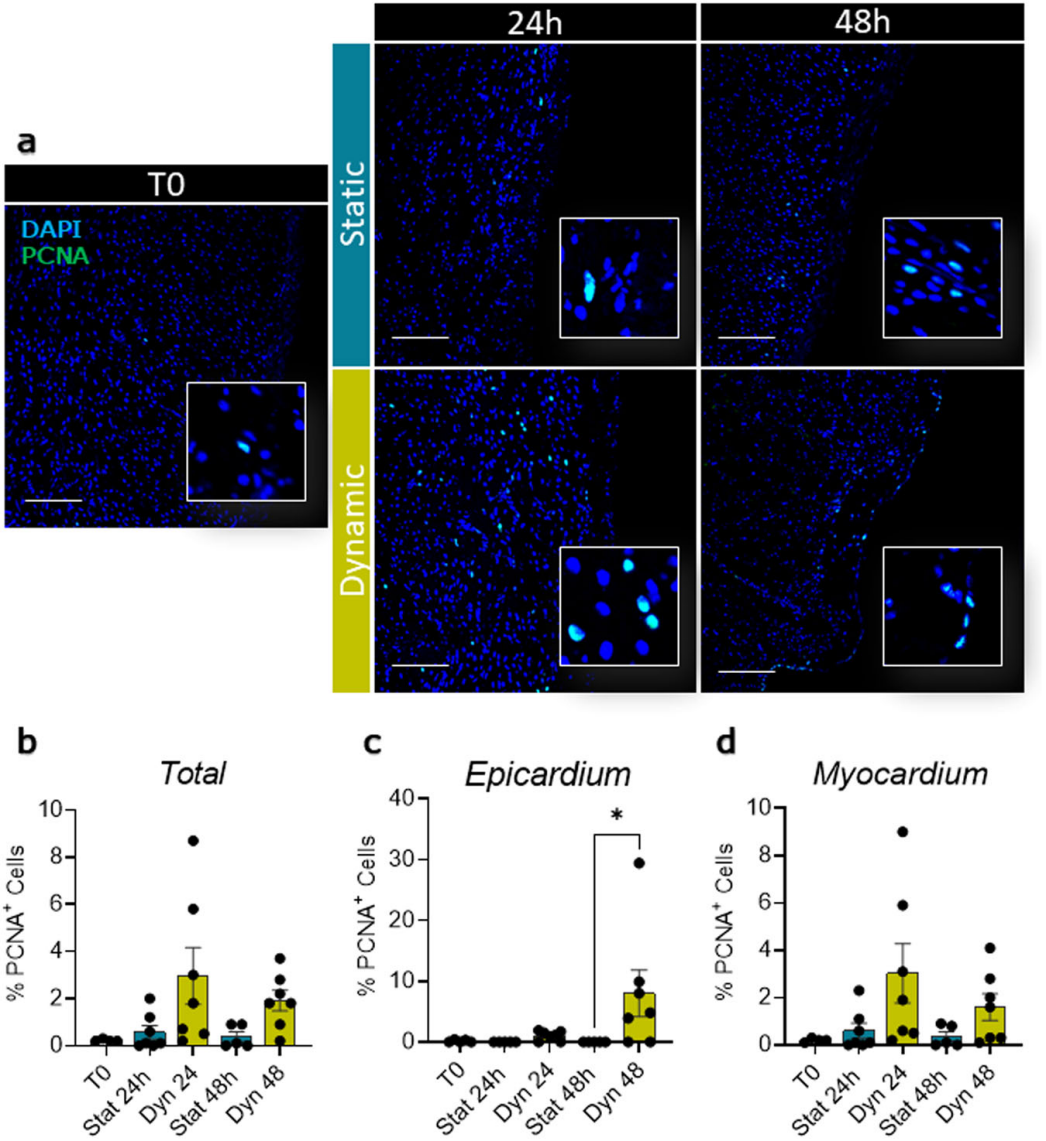
Dynamic slice culture increases epicardial cell proliferation. Proliferation was evaluated by PCNA antigen staining. Representative confocal images (**a**) and proliferating cell fraction in the whole slice (**b**), the epicardial (**c**), and the myocardial area (**d**) show no effect in static culture (Stat) and an increase following dynamic culture (Dyn). *N* of pigs = 5–6, number of slices displayed in graphs. All graphs display data as mean ± SEM. *p ≤ 0.05. Scale bar, 100 µm.

**Fig. 6:**
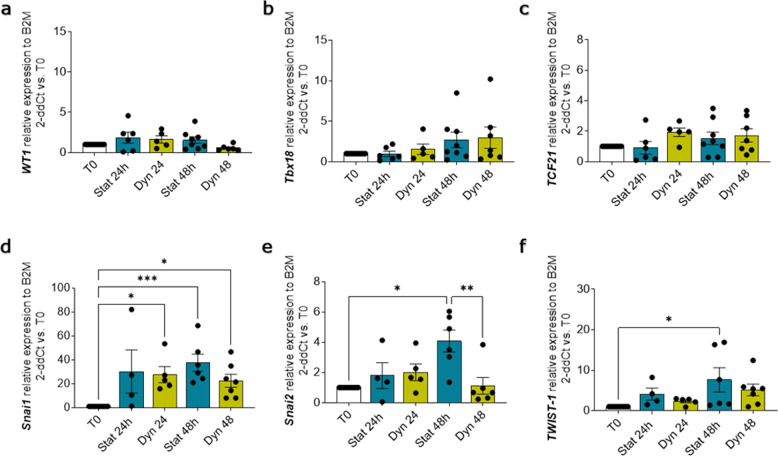
Epicardial and EMT gene expression in culture. Relative gene expression in the whole slice evaluated qPCR: expression of *WT1* (**a**), *Tbx18* (**b**), and *TCF21* (**c**) show no change in either static (Stat) or dynamic (Dyn) culture, relative to basal expression at T0. EMT markers *Snai1* (**d**) significantly increased in both culture conditions. *Snai2* (**e**) and *TWIST-1* (**f**) remained unchanged by the dynamic culture system but was upregulated in static when compared to freshly cut slices (T0). *N* of pigs = 3–7, number of slices displayed in the graph. All graphs display data as mean ± SEM. *p ≤ 0.05, **p ≤ 0.01, ***p ≤ 0.001.

Proliferating cell nuclear antigen (PCNA) staining in fresh slices, showed low basal proliferation levels for both the epicardium and myocardium (Epicardium 0.14 ± 0.08%; Myocardium 0.20 ± 0.04%, Fig. 5a), confirming the expected overall quiescent physiological state. This basal proliferation rate was maintained in static cultures, whilst the dynamic culture system stimulated moderate proliferation in the epicardial cells which was significantly increased at 48 h, as compared to static cultures (Dyn 48 h 8.00 ± 3.83% vs. Stat 48 h *p* = 0.0391, Fig. 5b–d). Slice culture did not intrinsically affect the expression of epicardial transcription factors *WT1*, *Tbx18*, and *Transcription factor 21* (*TCF21*), independently of the culture protocol (Fig. 6a–c), while the expression of the EMT marker *Snai1* appears to be upregulated in both. However, other EMT markers such as *Snai2* and *twist family bHLH transcription factor 1* (*TWIST-1*) were upregulated only after 48 h of static culture (Fig. 6d–f).

Taken together, these results indicated that epicardial slices can be successfully cultured ex vivo presenting minimal loss of viability in both static and dynamic culture. The constant oxygenation and circulation of nutrients and strict control of the pH afforded by the dynamic system additionally stimulated low levels of epicardial cell proliferation without affecting epicardial activation and maintained better myocardial tissue structure.

### Tβ4 increases epicardial viability and promotes robust activation and EMT

**Fig. 7:**
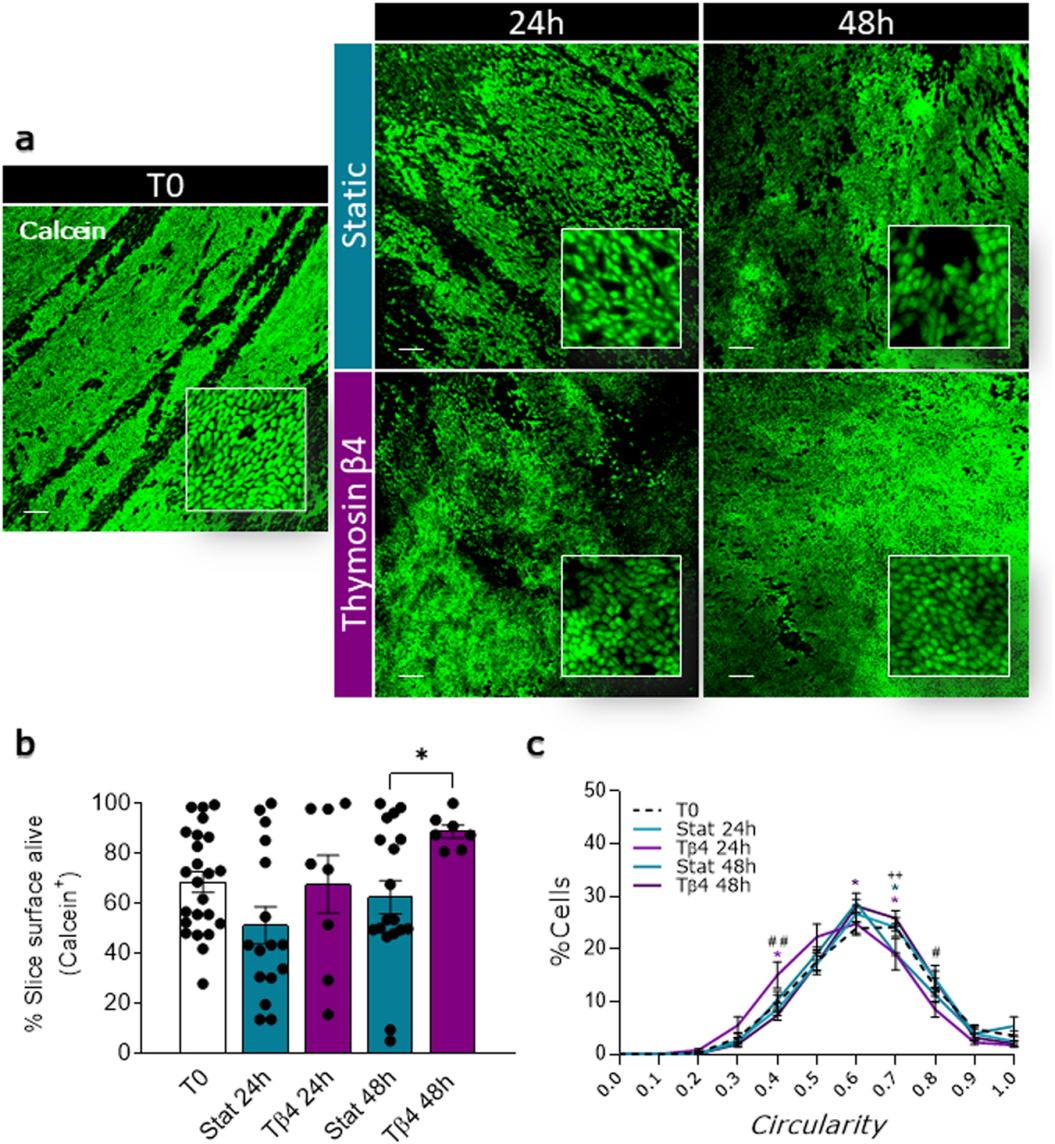
Tβ4 treatment alters epicardial cell viability and shape. Surface viability in freshly cut slices (T0), after 24 and 48 h of control static (Stat) culture and thymosin β4 (Tβ4) treatment (**a**, **b**). Representative confocal images (**a**) and quantification (**b**) indicates an increase in the percentage of live area upon Tβ4 treatment at 48 h. *N* of pigs = 4–14, number of slices displayed in the graph. Epicardial cells circularity distribution (**c**). *N* of pigs = 3–5, number of slices: T0 = 8, Stat 24 h = 5, Tβ4 24 h = 5, Stat 48 h = 5, Tβ4 48 h = 9. All graphs display data as mean ± SEM. *p ≤ 0.05 vs T0, ^#^*p* ≤ 0.05 Stat 24 h vs. Tβ4 24 h, ^##^*p* ≤ 0.001 Stat 24 h vs. Tβ4 24 h. ^++^*p* ≤ 0.001 Stat 48 h vs. Tβ4 48 h. Scale bar, 100 µm.

As the static culture system did not affect epicardial proliferation and due to the lower volume of medium required in this culture, we employed it as a model to assess the effect of the cardioprotective peptide thymosin β4 (Tβ4)^17^. Calcein AM staining showed that Tβ4 treatment further increased the viability of epicardial slices at 48 h, as compared to untreated cultures (Static 48 h 62.32 ± 6.70% vs. Tβ4 48 h 88.68 ± 2.57%, *p* = 0.0349, Fig. 7a, b). Furthermore, Tβ4 treatment while maintaining the overall characteristic cobblestone-like morphology of the epicardium (Circularity mean ± SEM: T0 0.673 ± 0.016; Stat 24 h 0.661 ± 0.017; Stat 48 h 0.669 ± 0.013; Tβ4 24 h 0.618 ± 0.019; Tβ4 48 h 0.677 ± 0.009), appeared to induce a decrease in circularity at 24 h (Fig. 7c). Data showed a shift in the distribution of circularity for Tβ4 at the 24 h time point, with a decrease in highly circular cells (0.8 score) and a significant increase in asymmetric cells (0.4 score) (Circularity 0.8: Stat 24 h 14.26 ± 2.59, Tβ4 24 h 8.50 ± 1.52, *p* = 0.0218. Circularity 0.4: Stat 24 h 8.56 ± 1.22, Tβ4 24 h 15.07 ± 2.50, *p* = 0.0065), potentially indicating differentiation.

**Fig. 8:**
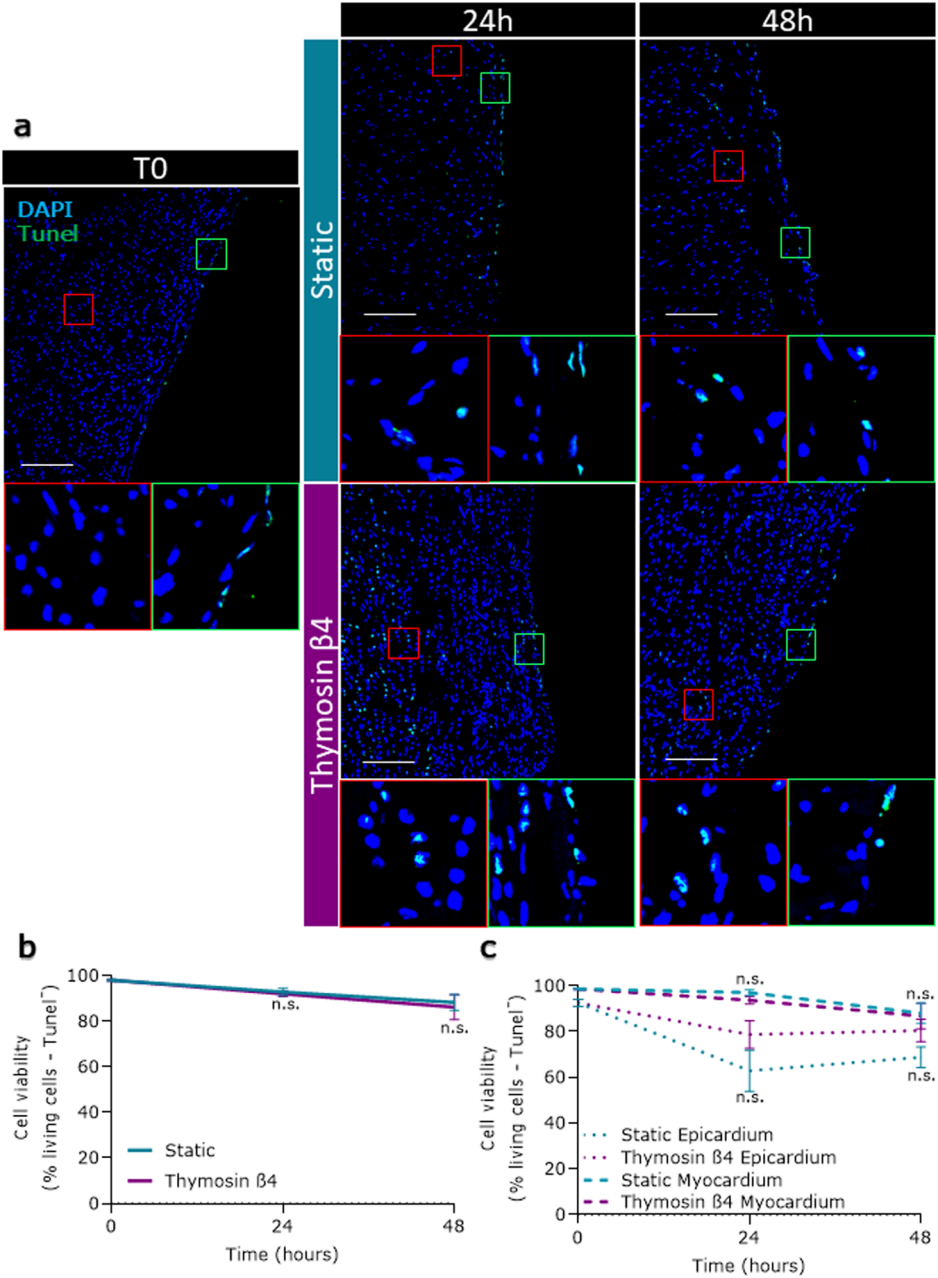
Apoptosis is unaffected by Tβ4 treatment. Apoptotic cells were evaluated by ApopTag detection kit assay (**a**); graphs report the fraction of non-apoptotic cells as a percentage of the total nuclei in the slice (**b**), and independently calculated fraction for myocardium and epicardium (**c**). *N* of pigs = 3–6, number of slices displayed in the graph. All graphs display data as mean ± SEM. n.s. not significant vs. Static control. Scale bar, 100 µm.

**Fig. 9:**
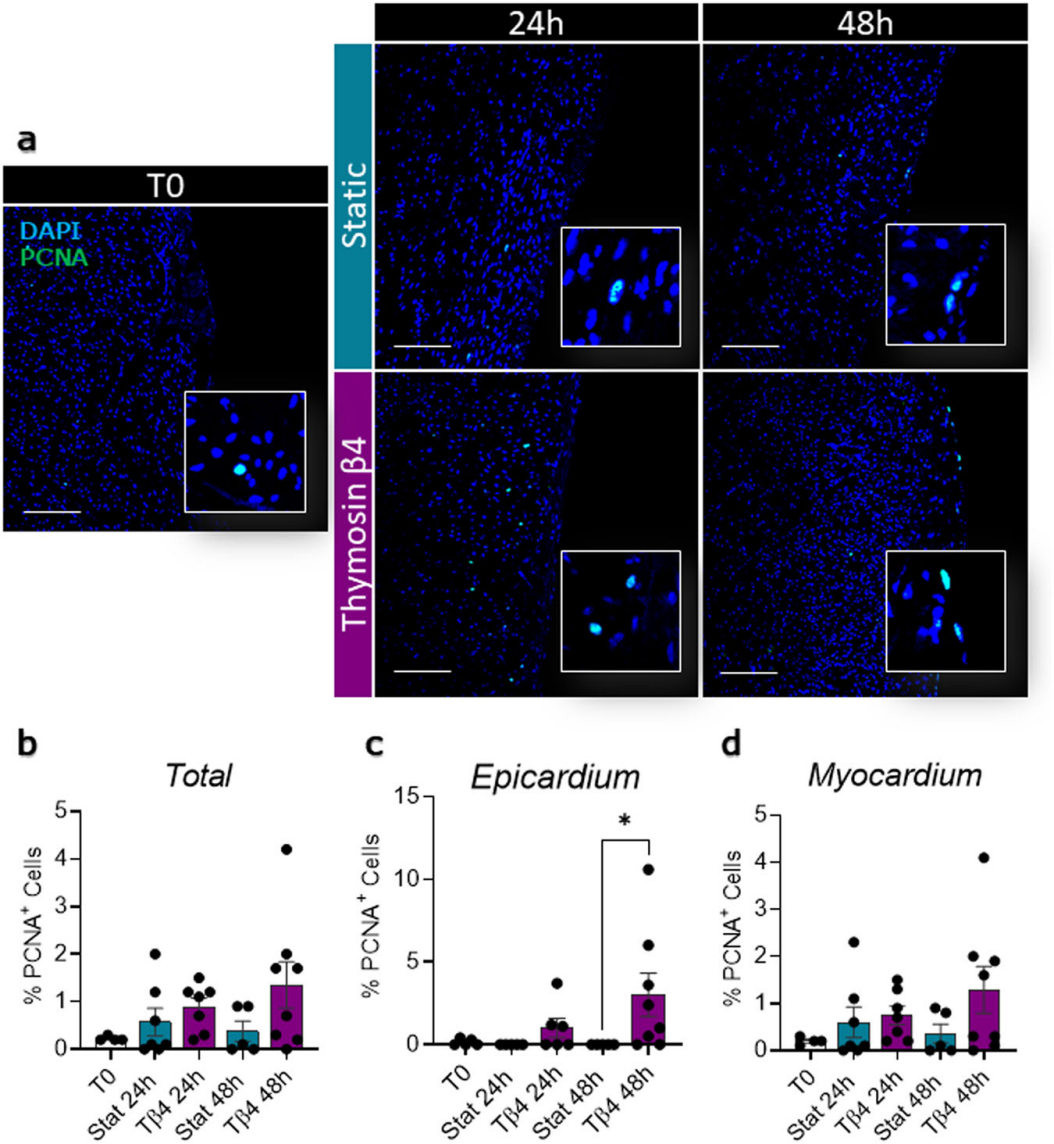
Percentage of proliferative cells assessed by PCNA staining. Representative images of the PCNA expression in freshly cut slices (T0), and after 24 and 48 h of control static culture (Stat) and thymosin β4 (Tβ4) treatment (**a**). Quantification of the percentage of proliferative cells normalized on total nuclei, in the whole slice (**b**), in epicardial (**c**), and myocardial area (**d**). *N* of pigs = 5–6, number of slices displayed in the graph. All graphs display data as mean ± SEM. *p ≤ 0.05. Scale bar, 100 µm.

**Fig. 10:**
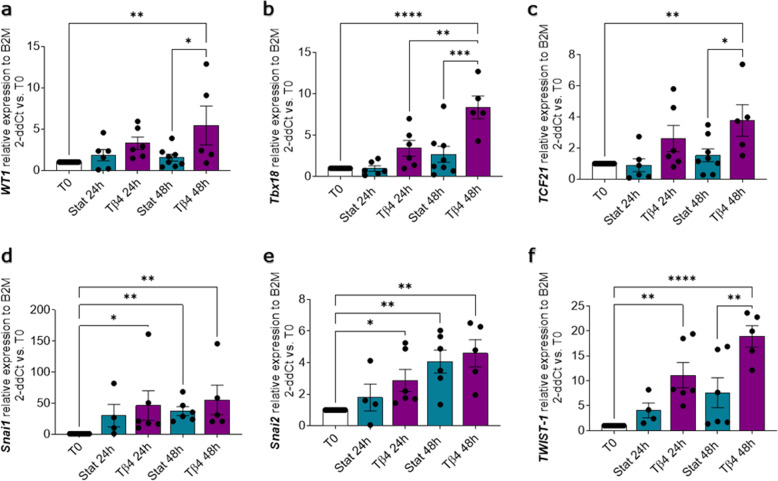
Tβ4 stimulates epicardial gene expression and EMT in the slices. Gene expression analysis showing relative expression of epicardial transcription factors *WT1* (**a**), *Tbx18* (**b**), and *TCF21* (**c**) and EMT markers *Snai1* (**d**), *Snai2* (**e**), and *TWIST-1* (**f**), after 24 h and 48 h of control static culture (Stat) and thymosin β4 (Tβ4) treatment, as compared to freshly cut slices (T0). *N* of pigs = 3–7, number of slices displayed in graphs. All graphs display data as mean ± SEM. *p ≤ 0.05, **p ≤ 0.01, ***p ≤ 0.001, ****p ≤ 0.0001.

Apoptosis in the whole slice, myocardium, and epicardium was unaffected by Tβ4 treatment (Fig. 8a–c). Proliferation was not significantly induced in the whole slice and the myocardium (Fig. 9a–d), but showed a significant increase in the epicardial layer (Fig. 9c). Simultaneously, Tβ4 treatment upregulated three to sixfold the expression of epicardial transcription factors *WT1*, *Tbx18*, and *TCF21*, as compared to the untreated control slices (Fig. 10a–c). For comparison, the expression of these transcription factors in myocardial slices was lower and not significantly affected by neither culture nor Tβ4 supplementation (*data not shown*). We also observed a strong overexpression of both Snails transcription factors (*Snai1* and *Snai2*) (Fig. 10d, e) and the significant upregulation of *TWIST-1* when compared to both T0 and untreated slices (11-fold at 24 h and 18-fold at 48 h, Fig. 9f), which suggests a Tβ4-dependent activation of the EMT pathways.

Tβ4 treatment protected the viability of the epicardium within the slice and specifically promoted epicardial cell proliferation. Moreover, Tβ4 treatment produced changes in epicardial cell shape and expression of genes related to activation and EMT differentiation.

### Tβ4 alters WT1^+^ cells distribution and induces differentiation

**Fig. 11:**
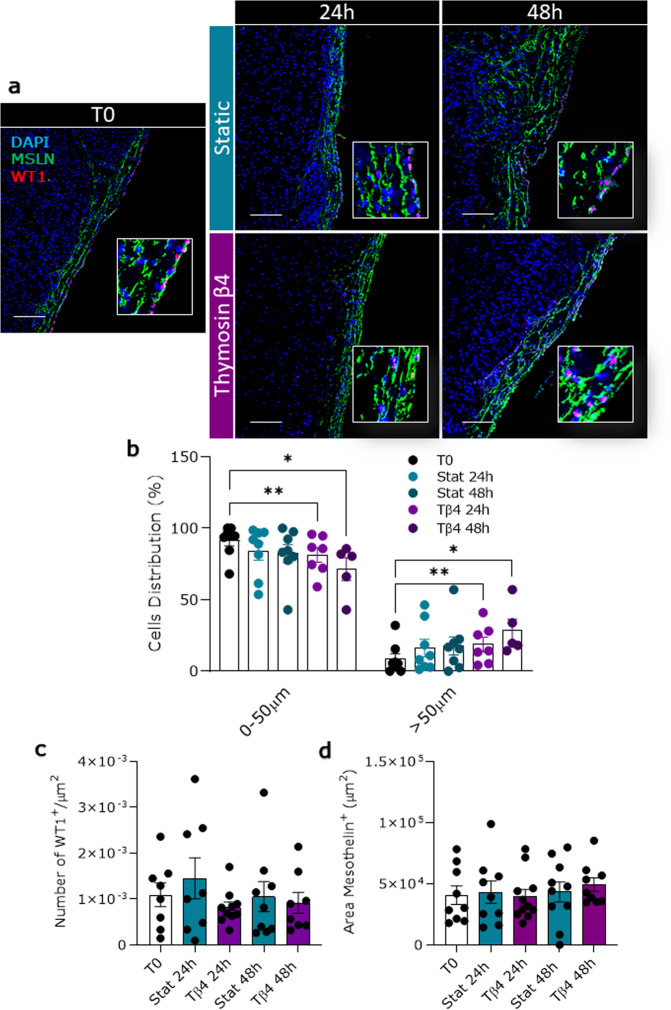
Tβ4 modifies WT1^+^ epicardial cell distribution in the sub-epicardial layer. WT1^+^ epicardial cells distribution evaluated as the average distance from the epicardial monolayer. Representative images of the distribution of WT1 and MSLN expression at different time points in freshly cut slices (T0) and after 24 and 48 h of control static (Stat) culture and thymosin β4 (Tβ4) treatment (**a**). Graph indicating the percentage of WT1^+^ cells retained in the epicardial layer (0–50 µm) or present in the area beneath the epicardium (>50 µm) in different culture conditions (**b**). Graph indicating the quantification of WT1^+^ cells in the whole slices (**c**). Graph indicating the area of mesothelin in the whole slice (**d**). *N* of pigs = 5–7, number of slices displayed in graphs. All graphs display data as mean ± SEM. *p ≤ 0.05, **p ≤ 0.01. Scale bars, 100 µm.

Activation of epicardial cells increases their migratory capacity and differentiation potential^18^. To estimate the reactivation of epicardial cells in our ex vivo model, we measured the localization of WT1^+^ cells within the MSLN^+^ layer, with respect to the heart surface. In freshly prepared slices, WT1^+^ cells were largely confined to the surface, in the epicardial layer (T0: 0–50 µm 91.51 ± 3.77%, >50 µm 8.48 ± 3.77%, Fig. 11a, b). Static culture did not affect this distribution, whilst Tβ4 treatment progressively decreased the number of WT1^+^ cells in the epicardial layer starting at 24 h (0–50 µm: Tβ4 24 h 81.24 ± 5.03% vs. T0, *p* = 0.0089), with a further reduction at 48 h (0–50 µm: Tβ4 48 h 71.42 ± 7.85% vs. T0 *p* = 0.0129, Fig. 11a, b). On the other hand, the percentage of WT1^+^ cells found in the sub-epicardial space increased in a time-dependent fashion (>50 µm: Tβ4 24 h 18.35 ± 5.03% vs. T0, *p* = 0.0089; Tβ4 48 h 28.58 ± 7.85 vs. T0, *p* = 0.0129, Fig. 11a, b). Importantly, the total number of WT1^+^ cells and the thickness of the MSLN layer in the whole slice remained overall unchanged by culture and treatments, indicating that the redistribution of WT1^+^ cells after Tβ4 treatment likely results from an increase in cell motility (Fig. 11c, d).

**Fig. 12:**
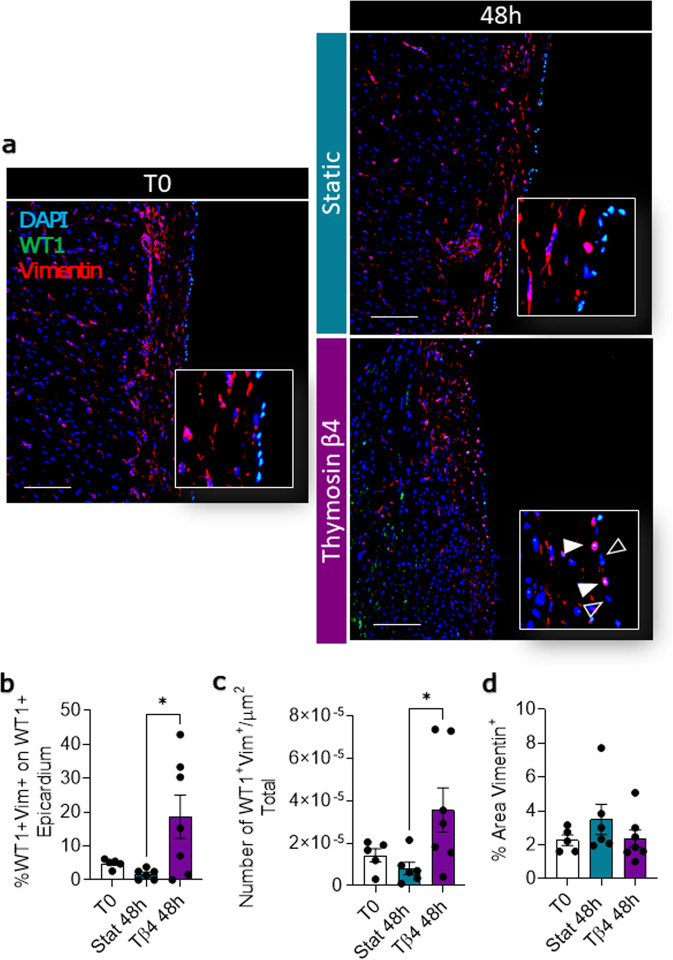
Tβ4 increases the differentiation into epicardial-derived mesenchymal cells. Representative images of WT1^+^/Vimentin^+^ cells in freshly cut slices (T0) and after 48 h of static (Stat) and thymosin β4 (Tβ4)-treated culture (**a**). Quantification of double-positive cells in the epicardial layer (**b**), and in the whole slice (**c**). Graph showing the quantification of vimentin area in the whole slice (**d**). White arrowheads indicating WT1^+^/Vimentin^+^ epicardial cells, empty arrowheads indicating WT1^**−**^ epicardial cells. *N* of pigs = 4–5, number of slices displayed in graphs. All graphs display data as mean ± SEM. *p ≤ 0.05. Scale bars, 100 µm.

Tβ4 has been described as a potent stimulator of epicardial mobilization, promoting differentiation into a variety of distinct phenotypes, including smooth muscle cells, fibroblast, and endothelial cells^18^. While we were unable to track epicardial cell differentiation into the slice due to the loss of WT1 marker, we investigated the differentiation of epicardial cells into epicardial-derived mesenchymal cells by quantifying the acquisition of the marker vimentin by WT1^+^ cells. In freshly isolated slices, WT1^+^/Vimentin^+^ cells were relatively infrequent, and this pattern was maintained in untreated static culture (Fig. 12a). However, treatment with Tβ4 significantly increased the percentage of double-positive cells both in the epicardial layer (T0 4.85 ± 0.59, Stat 48 h 1.72 ± 0.62, Tβ4 18.67 ± 6.39; Stat 48 h vs. Tβ4 48 h, *p* = 0.0377, Fig. 12b) and in the whole slice (Fig. 12c). The overall number of Vimentin^+^ cells within thec (Fig. 12d). To verify the bona fide epicardial origin of the WT1^+^ cells, we repeated the WT1/Vimentin staining on Tβ4 treated myocardial slices after 48 h of culture. Results showed an extremely limited presence of the WT1^+^ cells within the myocardial slices (Supplementary Fig. 3).

Taken together, these data indicated a Tβ4-dependent activating effect on WT1^+^ epicardial cells, leading to increase motility within the epicardial layer and differentiation into epicardial-derived mesenchymal cells. These results also supported the efficacy of this ex vivo model in enabling the study of WT1^+^ cell localization within the epicardium/myocardium interface and their differentiation into epicardial-derived mesenchymal cells.

## Discussion

The epicardium has emerged as one of the most relevant reservoirs for a comprehensive and coordinated regeneration of cardiac tissue^27^. Mouse studies demonstrated remarkable epicardial reactivation following apex amputation^28^ and heart ischemia^29^, but the reparative capacity of the epicardium is limited to early life and decreases dramatically in adulthood. In larger mammals, the paucity of representative models of heart remodeling limits the acquisition of translational understanding^29^. Currently, there is an unmet need for strategies to stimulate the epicardial cell activation and unlock their regenerative capacity, hence translating their promising experimental potential^30^ into therapeutic improvements^29^. Here, we describe a transformative organotypic 3D system representative of the epicardial/myocardial interface, containing all the cellular and extracellular components crucial to epicardial physiology.

Hitherto, myocardial slices from both animal and human sources have successfully enabled functional and pharmacological investigations into heart physiology^26,31,32^. The development of standardized protocols to obtain cardiac slices from small and large animals has allowed researchers to bridge the gap between in vitro and in vivo models, providing a valuable tool to study both the contractile and noncontractile cells of cardiac tissue^26,32^. However, typical myocardial slice preparation protocol requires the sacrifice of the epicardial layer, precluding the use of this valuable ex vivo model towards regenerative biology applications. Our preparation method was specifically designed to preserve the epicardium and the sub-epicardial region into the myocardium, in order to provide a functional model of the heart surface. To enable these preparations, we developed a unique embedding technique which efficiently protects the epicardium whilst maintaining the alignment of the cardiomyocytes. In fact, we utilized the flat surface provided by the epicardium to orientate the underlying heart tissue reducing damage during cutting, and obtaining myocardial viability similar to what was achieved in previous studies in myocardial slices^26,33^. In addition, we preserved the epicardial cells vitality and physiology by avoiding the practice of gluing the epicardium to the specimen holder^22,34^.

Epicardial slices retain heart tissue architecture in a comparable fashion with early reports in myocardial slices^26,32,35^, as demonstrated by the preservation of morphology and marker expression in the myocardial portion of the slice. More importantly, slices present an intact epicardial monolayer expressing typical markers such as MSLN, Uroplakin IIIB, E-cadherin, and WT1. Interestingly, about half of the epicardial cells on the surface of the porcine epicardial slice expressed WT1. For comparison, data in mice suggest that WT1^+^ cells account for over 90% of the epicardial cells in embryos; while in the adult lineage tracing studies report variable expression of WT1^8,36^. Although not entirely unique to the epicardial layer, this transcription factor has been extensively employed in lineage tracing studies in mice to identify epicardial progenitor cells^37,38^ and to describe their plasticity after injury^8,37^. The abundance of WT1^+^ cells in adult porcine epicardium suggests that a reservoir for regenerative cells exists in the heart of large mammals, encouraging the focus on new pharmacological strategies to promote their activation. In addition, epicardial cells and the immediate underlaying connective tissue strongly express MSLN, a surface marker characteristic of embryonic mesothelial progenitors originating fibroblasts and smooth muscle cells^39^. Studies in mouse hearts indicated that MSLN expression is limited to the superficial epicardial layer in these animals^8^. The widespread expression of MSLN across the epicardium and the sub-epicardial layer further support the existence of a sizable reservoir of epicardial progenitors in large adult mammal hearts. Comparison of our epicardial marker expression with results from other adults large mammal (including human) studies is hampered by the lack of available data. Indeed, our system enabled a comprehensive characterization of the epicardium in a large mammal heart, expanding our understanding of the comparative physiology of the epicardium.

We developed two separate protocols for the maintenance of epicardial slices in culture. The static culture system advances the current liquid–air interface approaches applied to myocardial slices^26^ by providing a pillared substrate which support the slice, maintaining the initial shape, and maximizing the contact surface area with the culture medium on both sides of the slice. We expect the tethering to the pillars to support the slice viability since previous reports suggest that the application of static mechanical forces to myocardial slices improves their performance in culture^22^. The dynamic culture system in addition uses a 3D printed insert that maintains a constant medium level during perfusion, connected to a feedback loop control system that monitors oxygen level and pH in the medium. Our results show that both culture systems preserve an unaltered cobblestone-shaped epicardial layer and sustain slice viability. Indeed, the overall calcein positive area fraction in our epicardial slices reflects previously reported data from myocardial slices which show a reduction of total viability around 20–40% after 2 days of culture^35,40^. In addition, the steadier environment provided by the dynamic culture system promoted the proliferation of epicardial cells and preserved structural organization.

Overall, the static culture represents a simpler, easily replicable system, that additionally enables small volume pharmacological studies. The dynamic system is tunable and allows finer control of culture conditions over time, and this may be advantageous for studies focusing on the effect of the epicardium on the myocardial physiology given the cardiomyocytes’ sensitivity to small changes in their environment, or in studies aiming at replicating pathological conditions (i.e., hypoxia/reperfusion).

To investigate the suitability of the epicardial slices for pharmacological and mechanistic studies, we treated the slices with Tβ4 and monitored the epicardial response. Previous studies in mice highlighted the potential therapeutic of Tβ4 after infarction, due to its ability to facilitate epicardial mobilization and promote neovascularization in the injured adult heart^18,41,42^. In our model, Tβ4 treatment further increases slice viability at 48 h of culture and epicardial cell proliferation, recapitulating previous data obtained in mice models of myocardial infarction^17^,^42^. In addition, Tβ4 treatment induces a shift in epicardial cell shape and a robust upregulation of *WT1*, *Tbx18*, and *TCF21*, a result that was specific to the epicardial slices. These transcription factors are characteristic of epicardial cells during embryonic heart development^8,43^, and are over-expressed upon injury^8^ and/or Tβ4 stimulation^9,44^, driving epicardial cell activation and contribution to cardiac repair^9,18,41^. In mice, the reparative process involves epicardial EMT and the subsequent invasion of the underlying myocardium, where the epicardial cells differentiates into various cardiac phenotypes^18,45–47^. Our model confirms the upregulation of the major epicardial EMT-inducible transcription factors, *Snai1*, *Snai2*, and *TWIST-1* in large animal epicardial slices upon Tβ4 treatment^7,48^. Concurrently, we detected a redistribution of WT1^+^ cells and an increased number of WT1^+^/vimentin^+^ cells within the epicardial layer. Importantly, the emergence of the WT1^+^/vimentin^+^ population was limited to the epicardial slices and did not occur in the myocardial slices, confirming the specific contribution of the epicardium. Of note, epicardial cells are heterogeneous in their marker expression, and their defining markers are also expressed by other cell populations within the heart, including vascular cells and fibroblasts^49^. For these reasons, despite a higher than normal ratio of epicardium to the myocardium in our slices and the comparison with the myocardial-only slices, we cannot completely exclude that overexpression of epicardial and EMT genes might be partially driven by other cell types.

Overall, these results confirm in large animal heart tissues the activating effects of Tβ4 treatment on epicardial cells that were previously described in vitro^18^, and in vivo in mice^8,9,16,42,50^. In addition, Tβ4 stimulation also increased the expression of vimentin in WT1^+^ cells, indicating differentiation into epicardial-derived mesenchymal cells. However, the lack of lineage tracing tools currently limits our capacity to draw conclusions on the fate of epicardial cells upon further differentiation, due to the loss of WT1 expression. Future work will focus on developing techniques aimed at permanently tagging the epicardial cells to track their migration within the slices.

Epicardial slices represent a ground-breaking tool for the study of epicardium activation within its natural microenvironment. By implementing this organotypic model it is possible to obtain up to 15–20 slices from a piglet heart, which might lead to a reduction of the number of small animals used in cardiovascular research. Additionally, this ex vivo model allows to study the effect of a treatment on epicardial, myocardial, and noncontractile cells of the heart at once. However, slices are characterized by relatively short-term viability in culture, as compared to classic in vitro models. This could represent a limitation when mimicking chronic disease onset. Furthermore, the implementation of physical stimuli, such as mechanical load or electrical stimulation, on the epicardial slices would improve the robustness of the model and warrants additional studies. In addition, epicardial slices lack the contribution of circulating inflammatory cells which play an important role in heart repair. On the other hand, removing the interference of the immune cells might help dissect the intrinsic repair mechanisms. Inflammatory stimuli can also be added to the culture media as in a normal in vitro experiment, helping to identify specific mediators.

We envision this model to be of use to researchers focusing on pharmacological and genetic strategies to activate the epicardium by providing a relatively high throughput platform based on physiologically relevant large animal tissue, to test initial candidates and observe epicardial proliferation, migration, and differentiation. Hypoxia and nutrient depletion can be easily implemented in our system thanks to the tunability of culture conditions, to mimic experimental myocardial infarction.

In conclusion, our study showed that living epicardial slices can be obtained from porcine hearts and cultured effectively under different conditions. We also demonstrate that this epicardial slice model can investigate epicardial cell activation. The ex vivo model we developed preserves the native microenvironment of the epicardium and provides control of monitored and adjustable culture conditions, laying the path for the development of patient-relevant systems using human-derived slices.

## Methods

### Experimental design

This study aimed to develop an ex vivo 3D organotypic model of the epicardial/myocardial interface, which would enable studies directed at identifying mechanisms of adult epicardium reactivation. Our experiments verified the maintenance of the tissue architecture in the slices and the preservation of a living and healthy epicardial cells monolayer and myocardial tissue. Following Tβ4 stimulation, we evaluated in situ the upregulation of epicardial embryonic genes, and the EMT, migration and differentiation of the WT1^+^ cells.

### Tissue samples

Swine hearts were obtained from 4–6 weeks old piglets, from The Pirbright Institute, (Pirbright, UK), and 16–20 weeks old pigs from the Newman’s Abattoir (Farnborough, UK). Animal experiments were carried out under the Home Office Animals (Scientific Procedures) Act (1986) (ASPA) and approved by the Animal Welfare and Ethical Review Board (AWERB) of The Pirbright Institute. The animals were housed in accordance with the Code of Practice for the Housing and Care of Animals Bred.

Pigs of 4–6 weeks were euthanized by an overdose of 10 ml pentobarbital (Dolethal 200 mg/ml solution for injection, Vetoquinol UK Ltd). All procedures were conducted by Personal License holders who were trained and competent and under the Project License PPL70/8852. After exsanguination, the thorax was opened using a sterile scalpel, and a transversal incision of the sternum allowed to open the chest and access the mediastinum. The hearts were rapidly collected after transection of the great vessels, maintaining the pericardium membrane intact, and then immediately submerged in 300 ml of ice-cold cardioplegia solution^23^ (NaCl 110 mM; CaCl_2_ 1.2 mM; KCl 16 mM; MgCl_2_ 16 mM; NaHCO_3_ 10 mM; pH 7.4), to remove the excess of blood. Retrograde heart perfusion (Fig. 1, step 1) was performed within 1 min from the excision using a 100 ml sterile syringe full of ice-cold cardioplegia connected to a three-way valve equipped with a 0.5 cm luer. The luer was inserted in the aorta and secured in position with a nylon cable tie. The cardioplegia was slowly injected into the heart, any bubble in the solution was removed by revolving the valve to direct the flow towards the open end. Effective flushing of the residual blood from the heart vessels was verified visually, before opening the pericardial membrane. Ventricles were removed by cutting along the left anterior descending artery and the posterior descending artery and immersed in ice-cold cardioplegia.

Abattoir pigs were euthanized according to local regulations. Abattoir pigs’ hearts were harvested from farm pigs at the abattoir and immediately washed with 500 ml of ice-cold cardioplegia, on-site. Ventricles were removed cutting along the left anterior descending artery and the posterior descending artery and then put in a hermetic plastic box with fresh ice-cold cardioplegia (800–1000 ml).

Independently from the source, samples submerged in ice-cold cardioplegia were packed in an insulated polystyrene box filled with cooler packs and transported to the lab. Tissue was only removed from the solution at the time of cutting (1–3 h from collection).

### Slice preparation

Before the epicardial slice preparation procedure may commence, the following needs to be completed: preliminary vibratome set up, preparation of embedding solution, embedding area staging, and recovery bath assembling.

The high precision vibratome (Leica, VT1200S) used for sectioning the tissue was cleaned with 70% ethanol and then distilled water. A double edge razor blade (Wilkinson Sword) was mounted onto the blade holder. Once in place, we checked the optimum positioning of the blade using Leica’s VibroCheck according to the manufacturer’s instructions. This allowed to minimize z-axis deflection of the blade during cutting at values comprised between −0.2 and 0.2 µm, avoiding tissue damages. The cutting amplitude was set at 1.5 mm and speed at 0.03 mm s^−1^. Next, the vibratome bath was mounted and filled with cold cutting/recovery solution (NaCl 140 mM; CaCl2 1.8 mM; KCl 6 mM; MgCl2 1 mM; Glucose 10 mM; 2-[4-(2-hydroxyethyl)piperazin-1-yl] ethanesulfonic acid (HEPES) 10 mM; 2,3-Butanedione monoxime (BDM) 10 mM; pH 7.4, 4 °C). The solution was bubbled with 99.5% oxygen for at least 30 min before starting to cut, and the outer part of the bath was filled with ice to maintain the temperature in the specimen bath.

The tissue embedding solution was prepared by dissolving 5% w/v of low melting agarose (ThermoScientific, TopVision Low Melting Point Agarose) in cutting/recovery solution, and microwaving briefly until completely melted. The embedding solution was left to cool down in a water bath set at 37 °C. The compliant surface for the alignment of the epicardial blocks consisted of a 0.5 cm thick agarose cushion. The cushion was made by dissolving 2% w/v of agarose (Invitrogen UltraPure Agarose) in a cutting/recovery solution, the liquid mix was poured into a Petri dish and allowed to solidify at room temperature before being placed on ice. For the tissue dissection and embedding area we used: a polystyrene box full of ice, a petri dish, and the agarose cushion already prepared, organized as in Fig. 1, step 2. Other necessary equipment were: single edge steel blades, anatomical forceps, 3D printed plastic ring, plastic Pasteur pipettes, and cyanoacrylate glue.

The recovery bath for the epicardial slices was prepared by placing a six-well culture plate with pierced bottom in a large plastic box filled with recovery solution at room temperature. In each well was placed a cell culture insert, and the cutting/recovery solution was bubbled with 99.5% oxygen continuously.

Once the low melting agarose solution was cooled at 37 °C, the dissecting area was prepared and the agarose cushion cooled down, it was possible to start the cutting procedure. The heart ventricle was placed on the Petri dish on ice (Fig. 1, step 3), tissue blocks 8 mm ×8 mm were dissected by making incisions through the full thickness of the ventricular wall with a single edge steel blade (Fig. 1, step 4). Ventricle cubes were placed on top of the agarose cushion with the epicardium facing down, inside a 3D printed mold. Embedding solution (5–8 ml) was gently poured on top with a Pasteur pipette (Fig. 1, steps 5–6). Once solidified, the embedded tissue cube was extracted from the ring and squared on one side to orientate the tissue on the specimen holder and facilitate the alignment with the blade (Fig. 1, step 7). The embedded tissue was then glued using cyanoacrylate glue onto the specimen holder, with the epicardium face up. The vibratome’s blade was carefully aligned to the top of the cube, setting the slicing start point. Next, 400–500 µm thick slices were cut (Fig. 1, step 8). During cutting, the sample was constantly submerged in cold and oxygenated cutting/recovery solution. After cutting, slices were incubated for at least 30 min in cutting/recovery solution at room temperature in the recovery bath (Fig. 1, step 9) before proceeding to culture or further analysis (Fig. 1, steps 10–11).

### Tissue culture

Slices were cultured epicardium-up on 8-mm-high Polydimethylsiloxane (PDMS) (SYLGARD 184) pillars cast at the bottom of a 100 mm petri dish (Fig. 1, step 10). Slices were held in places using entomology pins (A1 − 0.14 × 10 mm, Watkins & Doncaster). For static culture, the air–liquid interface was achieved by carefully adding culture medium (Medium 199 + 1X ITS Liquid Media Supplement + 1% Penicillin/Streptomycin Penicillin-Streptomycin + 10 mM of BDM -all Sigma-Aldrich) to leave the epicardium exposed to the atmosphere. This culture system maximized the contact between the myocardium and the culture medium. For epicardial cell reactivation experiments, the culture medium was supplemented with 100 ng ml^−1^ of Tβ4 (Human Thymosin beta 4 peptide, Abcam). For the dynamic culture, a custom-designed 3D printed adapter was inserted between the Petri dish and its lid providing inlet/outlet connection to the BioFlo 120 (Eppendorf) control station which provided a real-time feedback regulation of the medium pH at 7.4, oxygen saturation at 21% and provided a continuous flow rate of 4 ml/min. The level of the medium was precisely determined by the high of the inlet/outlet ports, maintaining a constant air–liquid interface. Slices were cultured in an incubator with humidified air at 37 °C and 5% CO_2_ for up to 48 h.

### Live staining

Slices were incubated at room temperature with 10 μM Calcein AM cell-permeant dye (Invitrogen, Thermo Fisher Scientific) for 45 min under continuous shaking. Following washes, confocal images were collected from three random fields of each slice using a ×10 objective on a Nikon Eclipse Ti A1-A confocal laser scanning microscope. Z-stack confocal images were generated from pictures taken at 5–10 µm intervals, 1024 × 1024 pixels, from 7 to 30 sections. The percentage of area stained was measured on maximum intensity projection images using ImageJ. Cell circularity was assessed on the epicardial cell-covered areas on maximum intensity projection images of the slices stained with calcein AM, using the ImageJ BioVoxxel plugin.

### Slice morphology

Slices were fixed in 4% PFA (Paraformaldehyde, Santa Cruz Biotechnology) overnight (o/n) at 4 °C, washed with phosphate buffer saline (PBS), and incubated overnight in 30% w/v sucrose (Sigma-Aldrich) solution in PBS. Slices were frozen embedded in OCT Compound (Agar scientific) in dry ice and cryostat sectioned longitudinally obtaining 5 µm thick sections.

Antigen retrieval was performed with microwave at 750 W for 15 min with citrate buffer (0.1 M Citric Acid, pH 6.0) or water bath at 80 °C for 30 min with tris-EDTA buffer (10 mM Tris Base, 1 mM EDTA Solution, pH 9.0) followed by permeabilization for nuclear antigens (0.1% Triton X-100 in PBS for 30 min) and blocked for 1 h at room temperature with 20% v/v Goat serum (Sigma-Aldrich) in PBS. Primary antibody incubation was performed overnight at 4 °C (WT1 1:50, E-cadherin 1:50, CD31 1:100, NG2 1:500 (water bath antigen retrieval performed) all from Abcam; Mesothelin 1:100 and Uroplakin IIIB 1:200 from Novus Biologicals; α-Actinin (Sarcomeric) 1:800, α-SMA 1:400, PCNA 1:100 all from Sigma-Aldrich; Connexin 43 1:300, Vimentin 1:100 all from Thermo Fisher Scientific), followed by the appropriate Goat anti-Mouse and/or Goat anti-Rabbit Alexa Fluor (Thermo Fisher Scientific) secondary antibody 488 and/or 567 diluted 1:200 for 1 h at 37 °C, and nuclei staining with DAPI (4′,6-diamidino-2-phenylindole, Merck) for 10 min at room temperature. Incubation with 0.1% Sudan Black (Sudan Black B, Santa Crus Biotechnology) in 70% ethanol w/v for 30 min at room temperature was performed to reduce tissue autofluorescence. Slides were then mounted in Fluoromount G (Invitrogen eBioscience Fluoromount G, Thermo Fisher Scientific) and imaged with Nikon Eclipse Ti A1-A confocal laser scanning microscope. Quantifications were performed on 3–7 random fields using ImageJ on 10x images.

### In situ detection of apoptosis and proliferation

For the detection of cell death in situ, we used the ApopTag kit (Merck) on 5 µm thick cryosections of fixed epicardial slices. Briefly, after rinsing the sections in PBS, equilibration buffer was applied on the specimens for 10 min at room temperature. TdT enzyme incubation was performed for 1 h at 37 °C, followed by an anti-digoxigenin conjugate solution for 30 min incubation at room temperature. Nuclei were counterstained with DAPI for 10 min at room temperature. Incubation with 0.1% w/v Sudan Black (Sudan Black B, Santa Crus Biotechnology) in 70% ethanol for 30 min at room temperature was performed to reduce tissue fluorescence background. Negative control was performed by omitting the TdT enzyme in the first incubation. As a positive control we used the same tissue sections pretreated with DNase I 3U/ml for 15 min at room temperature.

The proliferation of cells was evaluated by immunohistochemistry staining for proliferating cell nuclear antigen (PCNA). After antigen retrieval, accomplished with microwave at 750 W for 15 min with citrate buffer (0.1 M Citric Acid, pH 6.0), blocking of unspecific binding of the primary antibody was performed by incubating the slides with 20% Goat serum (Sigma-Aldrich) in PBS for 1 h at room temperature. Incubation with anti-PCNA Antibody clone PC10 (Merck), diluted at 1:100 was carried overnight at 4 °C. Signal detection was provided by goat anti-mouse Alexa Fluor secondary antibody 488 incubation, diluted at 1:200, for 1 h at 37 °C. Nuclei were stained with DAPI for 10 min at room temperature. Incubation with 0.1% Sudan Black (Sudan Black B, Santa Crus Biotechnology) in 70% ethanol (w/v) for 30 min at room temperature was performed to reduce tissue autofluorescence.

In both protocols, tissue slides were then mounted in Fluoromount G (Invitrogen eBioscience Fluoromount G, Thermo Fisher Scientific) and imaged with Nikon Eclipse Ti A1-A confocal laser scanning microscope. Quantifications were performed on 3-7 random fields using ImageJ on 10x images.

### Gene expression analysis

RNA was extracted using Promega reliaprep RNA Miniprep System (Promega) from homogenate tissues, and reverse-transcribed using QuantiTect Reverse Transcription Kit (Qiagen). Real-time PCR was performed on QuantStudio 7 Flex Real-Time PCR System (Applied Biosystems) using QuantiTect SYBR Green PCR Kit (Qiagen) and the primers in Supplementary Table 1 and results were normalized to the house-keeping gene β-2 microglobulin (B2M).

### Statistical analysis

Measurements were taken from distinct samples. Difference among groups were evaluated using one-way ANOVA or Kruskal–Wallis test, based on results from Shapiro–Wilk normality tests, followed by Fisher’s LSD post hoc test (GraphPad Prism 8.1.2). The difference between distributions of cell circularity was calculated with Kolmogorov–Smirnov test, using T0 as a reference distribution. Difference among groups within the same circularity score were evaluated using two-way ANOVA. A value of *p* < 0.05 was considered statistically significant. Data were presented as mean ± SEM.

### Reporting Summary

Further information on research design is available in the Nature Research Reporting Summary linked to this article.

## Supplementary information

Supplemental Figures and Table

Reporting Summary

## Data Availability

All raw data, images, and analyses supporting the findings of this study are available through the online repository Zenodo (10.5281/zenodo.5792892)^51^.
