# Supplementary material for: Epicardial slices: an innovative 3D organotypic model to study epicardial cell physiology and activation
Source: NPJ Regen Med. 2022 Jan 17;7:7. doi: 10.1038/s41536-021-00202-7 (PMC8764051; doi:10.1038/s41536-021-00202-7)
Supplement: Supplementary file 1 — Supplemental Figures and Table [file 41536_2021_202_MOESM1_ESM.pdf]

Supplementary Information

EPICARDIAL SLICES: AN INNOVATIVE 3D ORGANOTYPIC MODEL TO STUDY EPICARDIAL CELL PHYSIOLOGY AND ACTIVATION

Davide Maselli, Rolando Szilveszter Matos, Robert David Johnson, Ciro Chiappini, Patrizia Camelliti, Paola Campagnolo

Supplementary Figures

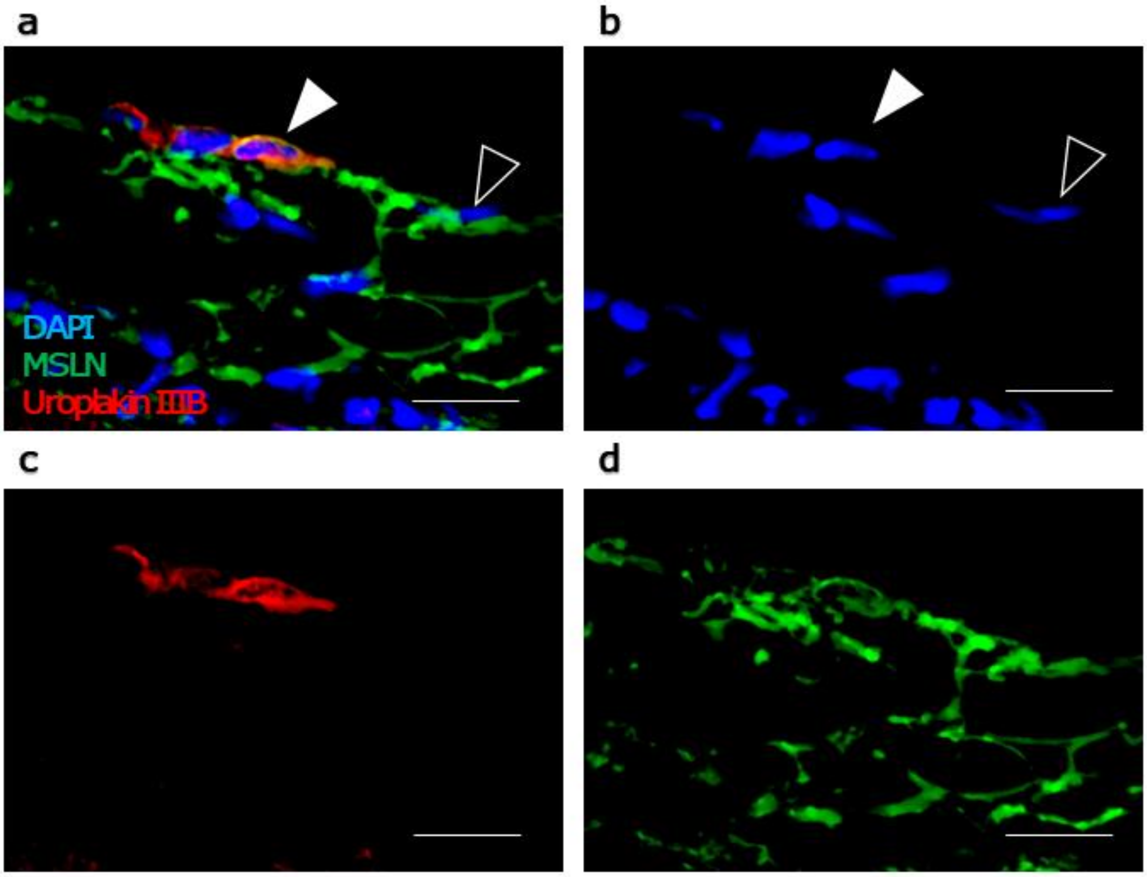

Supplementary Figure 1. Uroplakin IIIB expression in the epicardial layer. Confocal microscopy analysis of formaldehyde-fixed OCT-embedded porcine epicardial slices showing the merged picture (a), nuclear staining (DAPI, b), expression of the marker Uroplakin IIIB (c) and mesothelin (MSLN, d). Only a subset of mesothelin (MSLN)+ epicardial cells expressed Uroplakin IIIB. White arrowheads indicating Uroplakin IIIB+ /MSLN+ cell, empty arrowhead indicating Uroplakin IIIB- epicardial cell. Scale bars, 20µm.

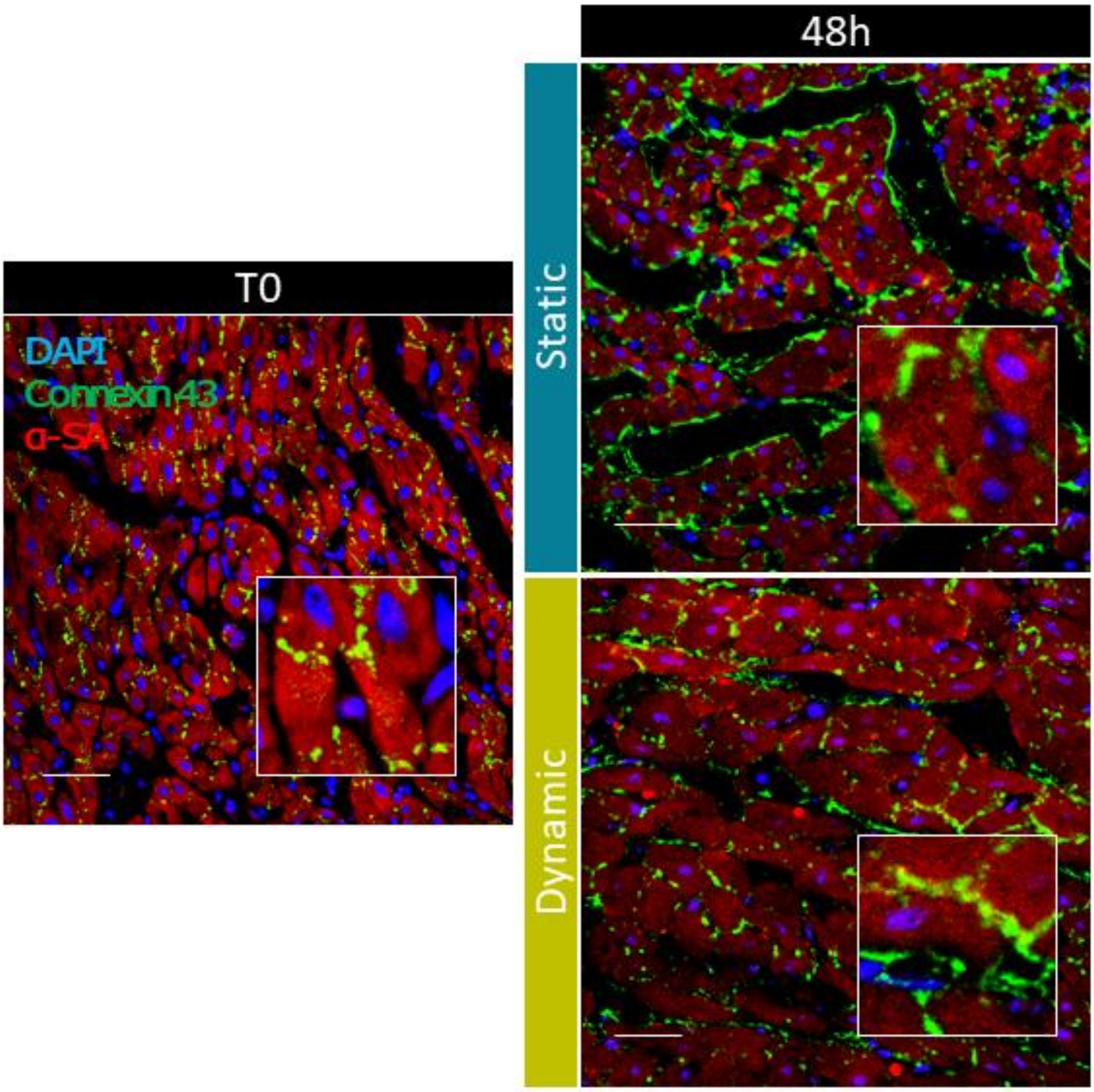

Supplementary Figure 2. Sarcomeric structure of myocardium after 48h of culture. Confocal microscopy analysis of formaldehyde-fixed OCT-embedded porcine epicardial slices showing cardiomyocytes beneath the epicardial layer displaying α-sarcomeric actin structures and connexin 43 within the gap junctions. Scale bars, 50µm.

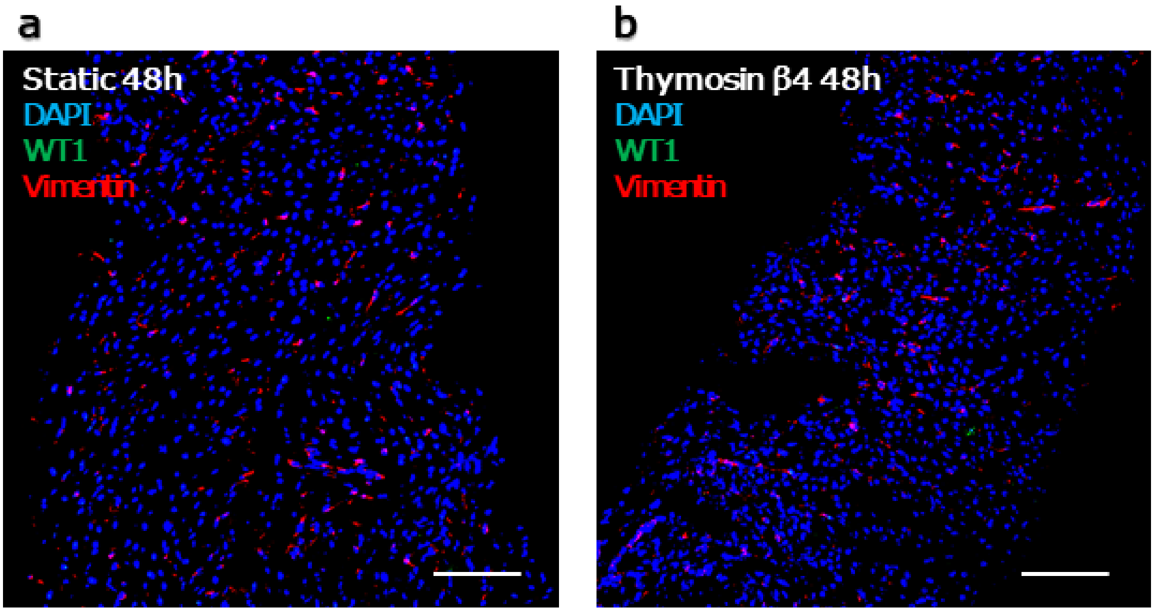

Supplementary Figure 3. Myocardial slices do not contain WT1+ /vimentin+ cells after 48h of culture. Representative images of WT1/Vimentin double-staining at 48h of static (a) and Tβ4-treated culture of myocardial slices (b). WT1 staining is undetectable in the tissue. Scale bars, 100μm.

Supplementary Table

Gene	Sequence (5'-3')
B2M	AAACGGAAAGCCAAATTACC
	ATCCACAGCGTTAGGAGTGA
WT1	TGAGCGAAGGTTTTCTCGTT
	GCTGAAGGGCTTTTCACTTG
Tbx18	GGGAGACTTGGATGCGACA
	GCGGTGACAGTGGTGAAAA
TCF21	GGAGTGTGAGACCCAACCA
	AGTCCAGCATCTCCACCTCT
Snai1	TTGCTGACCGCTCCAACCTG
	GGAGCACGTCTTGCACTGGT
Snai2	CGAGCCTACAGCCCCATTGC
	CTTTCGGAGCCGCTGTGGTC
TWIST-1	GCCCGTGGACAGTGATTCCC
	AGTGGTGATTGGCACGACC

Supplementary Table 1. List of primers used in this study
