# Supplementary material for: Epicardial slices: an innovative 3D organotypic model to study epicardial cell physiology and activation
Source: NPJ Regen Med. 2022 Jan 17;7:7. doi: 10.1038/s41536-021-00202-7 (PMC8764051; doi:10.1038/s41536-021-00202-7)
Supplement: Supplementary file 2 — Reporting Summary [file 41536_2021_202_MOESM2_ESM.pdf]

nature portfolio

Corresponding author(s): Paola Campagnolo

Last updated by author(s): Jul 29, 2021

Reporting Summary

Nature Portfolio wishes to improve the reproducibility of the work that we publish. This form provides structure for consistency and transparency in reporting. For further information on Nature Portfolio policies, see our Editorial Policies and the Editorial Policy Checklist.

Statistics

For all statistical analyses, confirm that the following items are present in the figure legend, table legend, main text, or Methods section.

n/a	Confirmed	
☐	☒	The exact sample size (n) for each experimental group/condition, given as a discrete number and unit of measurement
☐	☒	A statement on whether measurements were taken from distinct samples or whether the same sample was measured repeatedly
☐	☒	The statistical test(s) used AND whether they are one- or two-sided<br>*Only common tests should be described solely by name; describe more complex techniques in the Methods section.*
☒	☐	A description of all covariates tested
☐	☒	A description of any assumptions or corrections, such as tests of normality and adjustment for multiple comparisons
☐	☒	A full description of the statistical parameters including central tendency (e.g. means) or other basic estimates (e.g. regression coefficient) AND variation (e.g. standard deviation) or associated estimates of uncertainty (e.g. confidence intervals)
☐	☒	For null hypothesis testing, the test statistic (e.g. F, t, r) with confidence intervals, effect sizes, degrees of freedom and P value noted<br>*Give P values as exact values whenever suitable.*
☒	☐	For Bayesian analysis, information on the choice of priors and Markov chain Monte Carlo settings
☒	☐	For hierarchical and complex designs, identification of the appropriate level for tests and full reporting of outcomes
☒	☐	Estimates of effect sizes (e.g. Cohen's d, Pearson's r), indicating how they were calculated

Our web collection on statistics for biologists contains articles on many of the points above.

Software and code

Policy information about availability of computer code

Data collection	NA
Data analysis	NA

For manuscripts utilizing custom algorithms or software that are central to the research but not yet described in published literature, software must be made available to editors and reviewers. We strongly encourage code deposition in a community repository (e.g. GitHub). See the Nature Portfolio guidelines for submitting code & software for further information.

Data

Policy information about availability of data

All manuscripts must include a data availability statement. This statement should provide the following information, where applicable:

- Accession codes, unique identifiers, or web links for publicly available datasets
- A description of any restrictions on data availability
- For clinical datasets or third party data, please ensure that the statement adheres to our policy

The data that support the findings of this study are available from the corresponding author upon reasonable request. Raw data will be made available through an online repository upon publication.

Field-specific reporting

Please select the one below that is the best fit for your research. If you are not sure, read the appropriate sections before making your selection.

☒ Life sciences ☐ Behavioural & social sciences ☐ Ecological, evolutionary & environmental sciences

For a reference copy of the document with all sections, see nature.com/documents/nr-reporting-summary-flat.pdf

Life sciences study design

All studies must disclose on these points even when the disclosure is negative.

Sample size	We expected differences between 20 and 40% in the slice viability and epicardial gene expression. With 10 and 20% standard deviation, respectively, n=4 should provide a Power (1-β)= 0.8 with α=0.05. All experiments were performed on 4 or more slices
Data exclusions	Datasets were tested for outliers by Grubbs' test, also called the ESD method (extreme studentized deviate).
Replication	Experiments were reproduced successfully in several samples from at least 3 different animals (often 6-8).
Randomization	Samples were randomly allocated to experimental groups
Blinding	Blinding was not possible as the same operator performed the sample preparation and analysis.

Reporting for specific materials, systems and methods

We require information from authors about some types of materials, experimental systems and methods used in many studies. Here, indicate whether each material, system or method listed is relevant to your study. If you are not sure if a list item applies to your research, read the appropriate section before selecting a response.

Materials & experimental systems

n/a	Involved in the study	
☐	☒	Antibodies
☒	☐	Eukaryotic cell lines
☒	☐	Palaeontology and archaeology
☐	☒	Animals and other organisms
☒	☐	Human research participants
☒	☐	Clinical data
☒	☐	Dual use research of concern

Methods

n/a	Involved in the study	
☒	☐	ChIP-seq
☒	☐	Flow cytometry
☒	☐	MRI-based neuroimaging

Antibodies

Antibodies used	Primary: E-cadherin, CD31, NG2 all from Abcam; Mesothelin and and Uroplakin IIIB from Novus Biologicals; α-Actinin (Sarcomeric), α-SMA , PCNA all from Sigma-Aldrich; Connexin 43 , Vimentin all from Thermo Fisher Scientific. Secondary: Goat anti-Mouse and/or Goat anti-Rabbit Alexa Fluor (Thermo Fisher Scientific) 488 and/or 567
Validation	Primary antibodies were tested in a range of concentrations as suggested on their manufacturer's manual, and using different unmasking strategies depending on the subcellular localization of .the protein of interest. As none of the antibodies were validated on porcine tissues, we choose the protocol combinations that revealed clear expression in known structures (for example blood vessels for CD31 and SMA), and absence of unspecific staining in the remaining tissue. In some cases, several antibodies were tested in parallel (i.e. NG2), and the ones that did not provide satisfactory results were discarded from further experiments.

Animals and other organisms

Policy information about studies involving animals; ARRIVE guidelines recommended for reporting animal research

Laboratory animals	The study did not involve procedures performed on laboratory animals. Porcine hearts were obtained from 2 sources: Schedule 1 culleg piglets from the Pirbright Institute (Pirbright,UK) and abattoir animals from the Newmans Abattoir (Farnborough, UK). No procedure was performed on live animals.
Wild animals	NA
Field-collected samples	NA

Ethics oversight	Schedule 1 culling was carried out under the Home Office Animals (Scientific Procedures) Act (1986) (ASPA) and approved by the Animal Welfare and Ethical Review Board (AWERB) of The Pirbright Institute.

Note that full information on the approval of the study protocol must also be provided in the manuscript.
